# Advanced Non-Destructive Ocular Visualization Methods by Improved X-Ray Imaging Techniques

**DOI:** 10.1371/journal.pone.0170633

**Published:** 2017-01-27

**Authors:** Christian Enders, Eva-Maria Braig, Kai Scherer, Jens U. Werner, Gerhard K. Lang, Gabriele E. Lang, Franz Pfeiffer, Peter Noël, Ernst Rummeny, Julia Herzen

**Affiliations:** 1 Department of Ophthalmology, University of Ulm, Prittwitzstrasse 43, 89075 Ulm, Germany; 2 Chair of Biomedical Physics, Departement of Physics and Institute of Medical Engineering (IMETUM), Technical University of Munich, James-Franck Strasse 1, 85748 Garching, Germany; 3 Department of Diagnostic and Interventionial Radiology, Technical University of Munich, Ismaninger Strasse 22, 81664 Munich, Germany; University of Florida, UNITED STATES

## Abstract

Due to limited X-ray contrast, the use of micro-CT in histology is so far not as widespread as predicted. While specific staining procedures—mostly using iodine—address this shortcoming, long diffusion times restrict its use in the often time-constrained daily routine. Recently, a novel staining protocol has been proposed using a biochemical preconditioning step, which increases the permeability of the cells for the staining agent. This could enable the imaging of entire organs of small mammals at a yet unmatched image quality with reasonable preparation and scan times. We here propose an adaptation of this technique for virtual ophthalmology and histology by volumetrically assessing both human and porcine eyes. Hereby, we demonstrate that (contrast-enhanced) micro-CT can outperform conventional histology in the assessment of tumor entities, as well as functioning as a supplementary tool for surgeons in the positioning of intraocular implants *in-vitro* and as a general assessment tool for ophthalmologic specimens.

## Introduction

Histopathological work-up using light microscopy is the current gold-standard in the assessment and evaluation of pathological changes at a microanatomical level. The preparation protocol includes embedding of the tissue into paraffin and subsequent micro-scale slicing of the specimen. However, it is well known that the underlying slicing process carries the risk of material loss and sample distortion, among others folding, detachment and dislocation of anatomical structures [1–3]. Furthermore, the specimen itself as well as the information carried by its geometric/volumetric properties are irreversibly lost during the slicing procedure. Especially in the field of biological basic research, three-dimensional reconstruction methods have been proposed to circumvent the aforementioned shortcomings [4–8]: volumetric data are reconstructed by step-wise assembling of serial image slices. Although reconstruction and alignment procedures have been optimized over the years, the underlying methods are not applicable to a clinical setting, as preparation is extremely time consuming [9, 10]. Further, the repeated slicing process is especially vulnerable to the accumulation of geometrical distortions and, not least the resulting image quality is low.

While a wide range of other micro-scale imaging methods are available, they are not suitable for generalized use within an ophthalmopathological routine. Ultra-high field magnetic resonance imaging microscopy at 7 Tesla for instance, provides images with high soft-tissue contrast of the eye, adjacent structures, and intraocular tumors with good correlation to histology [11, 12], however, underlying micro-MRI setups are very expensive, cannot be used with metal implants and the image resolution is technically limited to a few micrometers. In ophthalmology, CT has become a frequently administered diagnostic procedure in the work-up of orbital diseases, for example in the diagnosis of orbital fractures, the identification of foreign bodies or the vizualization of orbital swellings. Moreover, CT imaging is used to assess the positioning and technical integrity, biocompatibility and biostability of retinal implants [13]. Normal *in-vivo* eye dimensions can be measured with CT imaging [14] but the resolution of clinical CT systems is not sufficient to adress specific ophthalmologic questions and soft-tissue contrast is quite low.

However, X-ray micro-CT seems to be a more promising candidate for ophthalmic applications in virtual histology, providing a far greater resolution (down to sub-microns), whilst being also commercially available. Besides, micro-CT is a method which has been widely applied and intensively studied in material science, small animal studies and for the precise imaging of human tissue samples [15, 16].

In terms of experimental realization and handling, X-ray micro-CT systems are now standalone, highly automated and easy to handle also by non-trained users [17]. However, conventional micro-CT still has one major limitation: the variance of material density determining the contrast of X-ray absorbtion-based imaging is inherently low in ophthalmologic soft-tissue samples.

To overcome this limitation, various soft-tissue staining techniques have been proposed [18–20]. Hereby, the sample is loaded with high atomic-number stains which are selectively incorporated in the tissue cells, so that the local contrast is significantly enhanced. However, these techniques require long diffusion times and may cause sample shrinkage [21–23]. Recently, a novel staining procedure has been proposed, which overcomes aforementioned problems by applying a prior dehydration step to the specimen, allowing for a fast and isotropic diffusion of the stain into the sample and preventing shrinkage [24]. This method could allow for a uniform contrast enhancement even in larger samples, as for instance *ex-vivo* high-contrast imaging of entire small animal organs.

We here propose an adaptation of this technique for ophthalmologic settings, namely: the evaluation of tumor entities, as a supplementary tool for the assessment of the positioning of intracoular implants and the general assessment of ophthalmologic parameters. We are convinced that non-destructive (contrast-enhanced) micro-CT may serve as a valuable tool for clinical histology in near future.

## Materials and Methods

The study was conducted in accordance with the declaration of Helsinki and was approved by the institutional review board (Institutional review board of the University of Ulm, application number 100/15, approval received 29/07/2015). Written informed consent was obtained from the included patient. Fresh, non-processed porcine eyes were acquired from the local slaughter house (Fleischmarkt Donautal, Steinbeisstraße 17, 89079 Ulm, Germany).

### Specimen preparation

The porcine eyes (specimens I and II) were fixed in 4% formalin. Specimen I was used to evaluate the implantation of a micro-stent (Transcend Medical Inc., Menlo Park, California, USA) before its clinical application. The technique provides a minimal invasive surgery to restore suprachoroidal aqueous outflow in open angle glaucoma patients. The eye was embedded into a paraffin block which was sliced at a thickness of 10 mm.

Specimen II was cut into three pieces: optic nerve, lens and a posterior eye-tissue segment prior to imaging. Each part of specimen II underwent a novel staining procedure as described by Silva, J. *et al* [24]. The protocol includes a prior dehydration in a graded series of ethanolic solutions with 50, 70, 80, 90, 96 and 100% ethanol (in distilled water) with 1 hour of dwell time each prior to staining in 1% wt. I_2_ solution and subsequent washing with 1% pure ethanol. The graded dehydration step avoids sample shrinkage while additionally increasing the cell permeability for the staining agent.

The enucleated human eye (specimen III) underwent conventional clinical histopathological examination before it was provided for this study. Histological routine work-up includes fixation in 4% formalin, specific periodic acid-Schiff staining and subsequent embedding into paraffin. Thin slices of the sample block are cut with a microtome and imaged in transmitted light microscopy. The remaining paraffin block was then used for the microCT study without any further sample preparation.

### Micro-CT measurements

All samples were placed in plastic tubes and tomographic imaging was carried out with a VtomeX micro-CT system (General Electric, Measurement & Control Solutions, Little Chalfont, United Kingdom). It is equipped with a tungsten anode, which was operated at an acceleration voltage U_acc_ = 60kV and a filament current of I = 100μA for specimen I/II and I = 250μA for specimen II, respectively. The total measurement time (excluding the sample preparation time) was t = 1.5 h in case of specimen I/II and t = 5.5 h for specimen III, respectively, depending on the exposure time per projection and the total number of projections. Samples were positioned as close as possible to the X-ray source to achieve maximal magnification. By that an effective pixel-size between 10 and 27 μm can be achieved, depending on the specific sample size. Tomography data-sets were reconstructed internally with the implemented reconstruction tool and visualized using the commercial software Avizo (Fei, Hillsboro, Oregon). For more detailed information on the spatial resolution characterization of the used micro-CT see Ref. [25]

## Results

### Position control of a surgical implant (Case I)

In a first step, we used micro-CT to assess the exact position of a supraciliary microstent, which had been inserted into a procine eye during surgical training. Correct positioning of the microstent was assessed with conventional histology according to the usual practice, as described above.

**Fig 1:**
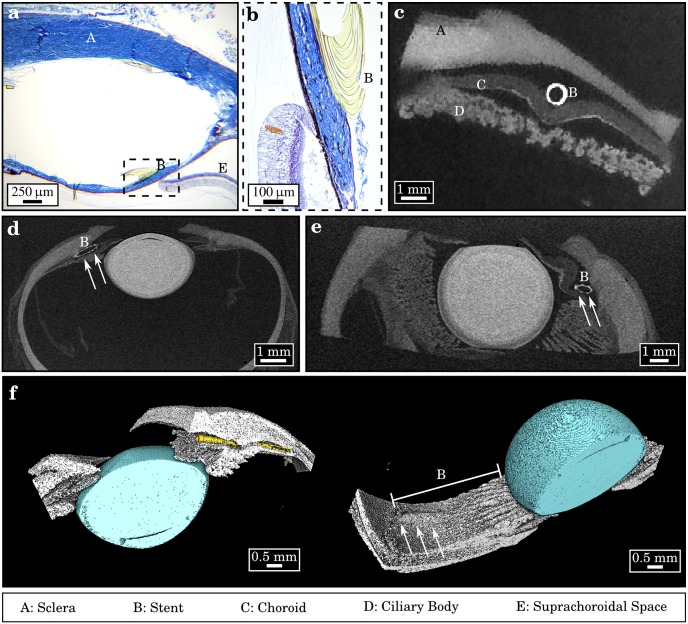
Position-control of an ophthalmic implant in conventional histology and in micro-CT imaging. (a) In the classical histological assessment, due to the slicing procedure, the stent was artificially broken. The former position could only be assessed by the remaining space and (b) remaining fragments in transmitted-light microscopy. (c) Transversal section of the reconstructed micro-CT imaging data-set demonstrated proper positioning of the stent in the supraciliary space. Sclera, outer choroid and the underlying ciliary body were easily distinguished by distinct gray-values. (d, e) Full length and penetration depth of the implant could only be visualized in transversal slices of the non-destructive micro-CT measurement. (f) X-ray contrast was sufficient to apply an automated material segmentation for a color-coded three-dimensional volume rendering. The position of the stent could here be evaluated without prior modifications to the sample.

Macroscopically the eye showed no pathologies and the stent was in place. In different histological slices, the entry points of the stent through the chamber angle could be identified. However, it was not possible to visualize the stent in its full length or to precisely determine its position, as sections of the latter had to be artificially broken during the slicing procedure, which is essential for the histological technique. Corresponding splinters of the stent were distributed along the chamber angle and the suprachoroidal space, appearing as yellow foreign bodies as shown in Fig 1a/1b.

In contrast, the micro-CT measurement of a second, equivalent eye was capable of revealing the precise position of the stent along its full length: within the various slices of the reconstructed volume (Fig 1c, 1d and 1e) the stent could be well distinguished as a white circle, being exactly situated within the suprachoroidal space, between sclera and choroid. Besides, different layers and structures of the eye bulb including the lens were clearly delimitated with different absorption properties, i.e. gray-values. Further, an evaluation of the surgical success could be carried out, since free lumen, depth of stent-penetration and a potential violation of surrounding tissue could be assessed. In this case no injured tissue was found. Finally, by using an automated material segmentation algorithm and volume-rendering (Avizo, Fei, Hillsboro, Oregon) the stent could be volumetrically assessed in its actual physiological constitution without prior modifications to the stent or the eye itself, which could have falsified position control (Fig 1f).

### Contrast-enhanced micro-CT (Case II)

In order to analyse the potential of contrast-enhanced micro-CT for general ophthalmologic questions, an optic nerve, a lens and a posterior eye-tissue segment of a porcine eye were assessed with this method.

**Fig 2:**
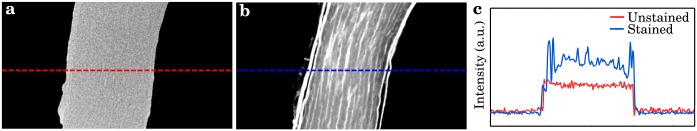
Contrast-enhanced micro-CT imaging. (a) Micro-CT section of an unstained and (b) an iodine-stained optic nerve. In the case of the unstained optic nerve (control) none of the anatomical substructures were distinguishable with micro-CT, since density variations within soft tissue are very low and thereby intrinsic X-ray contrast is small. Using a multiple-stage iodine staining procedure, the characteristic myeline-covered nerve fiber bundles, the septa as well as the dura mater, pia mater and the subarachnoid space became clearly discernable, due to a selective uptake of contrast-rich iodine. Both volumes were acquired with the same imaging parameters. (c) Corresponding line-plots of the intensity values through the tomographic slices visualizing a strongly enhanced overall and local contrast in case of the iodine-stained nerve. Both curves are not true to scale.

Diffusion of the high atomic-number staining agent into the tissue increased X-ray absorption depending on the permeability of the particular tissue type. As a result, the detail contrast was drastically enhanced compared to an unstained sample. Fig 2 shows an image section of a tomographic slice of the optic nerve alongside with respective line-plots before and after undergoing the aforementioned staining procedure. Within the unstained image slice (Fig 2a) the optic nerve yielded an insufficient contrast and thus appeared homogeneous, i.e. did not show any distinct micro-structure. Within the stained nerve (Fig 2b) a significant enhancement of small detail contrast was achieved, allowing for a clear depiction of dura and pia mater as well as multiple fine optic nerve fibers (described further below) as represented by the corresponding peaks in the line plot (Fig 2c).

**Fig 3:**
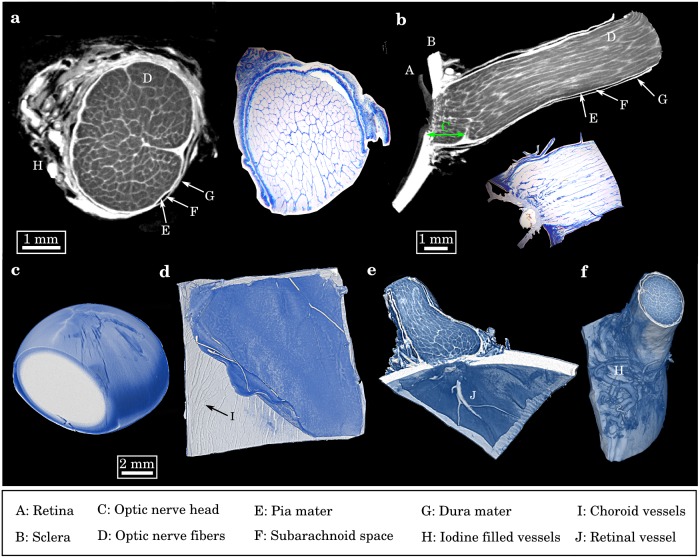
Anatomical assessment of ophthalmological structures using contrast-enhanced micro-CT imaging. (a) Transversal and (b) longitudinal slice through an iodine-stained porcine optic nerve, alongside with the corresponding histopathological sections in Azan-staining. Contrast-enhanced micro-CT imaging allowed a non-destructive depiction of all relevant anatomical features, i.e. myeline-coated, bundled nerve fibers, at quality comparable to lightmicroscopy at this resolution. (c) Micro-CT rendering of a porcine lens, showing a limited diffusion of staining agent into the interior. (d) Micro-CT rendering of a porcine eye tissue layer, showing the detachment of retina from subjacent choroid with vascularization. (e, f) Micro-CT rendering of a porcine optic nerve: at the optic disc the retinal ganglia cells exit and form the optic nerve. The vascularization of the nerve could be clearly identified by the iodine filled blood vessels.

Smooth penetration with the staining agent for the whole sample could be demonstrated in longitudinal (Fig 3a) and transverse tomographic slices (Fig 3b) of the optic nerve. High detail contrast allowed for the vizualization of the stent entry into the eye bulb through the lamina cribrosa. Besides, contrast-enhanced micro-CT allowed for vizualisation of sclera, retina, optic nerve fibers, pia mater, dura mater, subarachnoid space, in image quality and richness of detail comparable to histopathology (as shown in Azan-staining of an optic nerve).

Uptake of staining agent within the crystalline lens was poor, as expected. A corresponding volume rendering with clipping plane (Fig 3c) showed the limited diffusion depth of iodine for the lens which appears macroscopically transparent. Further, no signs of cataract were found.

Finally, a posterior section of eye tissue (Fig 3d) was investigated and showed an artificial retinal detachment in the volume-view. Hereby, a clear visualization of the underlying iodine filled choroidal vasculature was possible.

Microanatomical layers and structures could be identified (data not shown). From back to front: retinal vessels, retina, retinal epithelium, Bruch’s membrane, choroid and sclera.

In these volume reconstructions, high quality visualisation of the entire eye vascularization was achieved due to the enrichment of staining agent inside the vessels. The entrance point of the retinal vessels into the optic nerve (Fig 3e) and the vascularization alongside the nerve sheath (Fig 3f) were clearly distinguishable.

### Virtual histology of an enucleated human globe (Case III)

**Fig 4:**
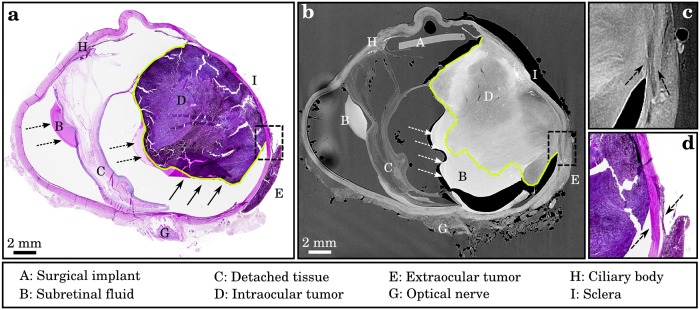
Virtual histology of an enucleated human globe. (a) Histopathological slice (PAS stain) and (b) correlated micro-CT of a human eye with malignant melanoma in the right half. Notice that the micro-CT section shows all medically relevant features, such as subretinal fluid (dotted black and white arrows), retinal detachment or the onset of the optic nerve, while enabling a better assessment of the exact tumor size in comparison to histopathology: the micro-CT image does not only map the posterior part of the tumor correctly (which is flipped over in b, as indicated by the black arrows), but also depicts an extra-ocular penetration of the tumor through retina, choroid and sclera (as indicated by dashed box). The density of the subretinal fluid and the intraocular tumor are similar to but still distinguishable as indicated by the green line. (c) Magnified section of the micro-CT image showing a continuous expansion (dashed arrows) of the extraocular tumor. In comparison, in the case of the corresponding histopathological section (d), this assessment is not feasible, since cancerous tissue is ripped.

In a last step, we investigated an enucleated human eye (unstained, embedded in paraffin), in order to evaluate the potential of micro-CT as a tool for virtual histology. Enucleation had been indicated in the donor because of a large malignant uveal melanoma. Macroscopically an extraocular extension of the melanoma was diagnosed. Histopathology (Fig 4a) revealed a malignant choroidal melanoma with ciliary body involvement and continuous extension into the sclera and extraocular extension. The melanoma was characterized by spindle B-cells, epitheloid cells, necrosis and pigmentation and was accompanied by retinal detachment and subretinal serous fluid. The optic nerve showed no signs of infiltration. The implanted artificial intraocular lens could not be examined microscopically because of the histologic slicing process. The anterior chamber was lost.

The micro-CT measurement was conducted without any further sample preparation, however it provided a distinct gray-value contrast for all relevant soft tissue components. We were able to freely chose the tomographic volume slice which corresponded to the histological slice enabling us to compare both methods (Fig 4b). The intra- and extraocular masses were identified as a heterogeneous, hyperdense structure in good agreement with histopathology. Alongside the surrounding tissue layers of the globe a retinal detachment and subretinal fluid were confirmed. The non-destructive character of the imaging method allowed for the evaluation of the geometrical configuration of the whole globe without any modification on the original sample conditions. In contrast, the histological slicing process caused subretinal fluid to be flipped up in the shown histology slice, as indicated by the arrows (Fig 4a). This complicated histological examination. In the reconstructed micro-CT volume the exact point of perforation where the tumor broke through the sclera was identified as shown in the enlarged section in Fig 4c, whereas this information was lost in histology (Fig 4d) due to the arbitrary choice of only a few slices. The subretinal fluid (Fig 4bB) showed homogenous density similar to but still distinguishable from the tumor mass (Fig 4bD). A correlation of the density in micro-CT with the measure of pigmentation or cell density in the tumor mass could not be found. All together, obtained micro-CT images showed a high correlation with histopathological findings and additionally, geometrical and volumetric information could be assessed faster and more accurately with the non-destructive high-resolution X-ray examination.

## Discussion

Within this study, we demonstrated the potential of micro-CT in an ophthalmology setting for visualizing normal anatomy, intraocular implants and pathological findings. First, we evaluated micro-CT as a helpful tool in the ex-vivo position control of a novel surgical implant, e.g. a measure to evaluate the results of operation in surgeon training. Within the histological slicing process the stent must be removed or will artificially be broken. The former position of the stent is then roughly approximated by assessing remaining cavities as well as stent-fragments in the surrounding tissue. Here, X-ray micro-CT provides an easy and much more precise method to image the surgical implant *in situ* without prior modification of the actual physiological conditions, which may alter the results. In addition, geometrical configuration data of the whole region of interest can be provided digitally, increasing accessibility and reproducibility of the findings. In addition, this method could substantially improve the assessment of procedures performed as part of ophthalmological surgical training, as the volumetric high-resolution visualization enhances the precision of evaluating the degree of success of ocular stent implantation in an animal model.

We pushed the boundries of micro-CT imaging even further, by demonstrating the feasibility of visualizing ocular microstructures and assessing potential histopathological findings. In comparison to the polyimide based micro-stent, which gives an inherently sufficient X-ray contrast, variation of X-ray absorption of soft tissues is small, which severely limits CT-image contrast between different kinds of soft tissue. The development of novel staining agents aims to counter this limitation. It has been shown that the application of a simple non-specific iodine staining can improve imaging contrast to the level of standard histological assessments. Even with a micro-CT imaging setup with moderate resolution, single nerve fibers and different tissue layers of the eye globe and optic nerve can be visualized with distinct gray-level values. The success of this simple approach could be a basis for further improvement of the staining method. The development of specific staining methods could enable specific diagnosis of different pathologies directly and further increase the spectrum of virtual histology.

In a last step we demonstrated the usability of this novel imaging technology in an ophthalmopathological routine setting, with a proof-of-principle measurement of an enucleated paraffin-embedded human globe. We found a high correlation with histological findings. Micro-CT imaging visualizes lesion-specific features and allows undistorted assessment of eye anatomy and ocular lesions while providing additional information on tumor expansion. Future studies are needed to evaluate the correlation of micro-CT density and histopathological intratumoral heterogenity. The handling of commercial micro-CTs is user friendly. For example for this measurement no further sample processing, especially no staining was required, enabling an easy integration of this method into a ophthalmopathological work-flow. The measurements were done with a whole paraffin block so that conventional histology would not be affected by previous micro-CT measurements. Here we cannot exclude that the X-ray contrast for the sample even profits from the dehydration during histological embedding resulting in the seen high soft-tissue contrast. We were able to show that three-dimensional measurement provided additional diagnostic information about the geometrical configuration of the whole sample without producing the usual distortions arising from the histological slicing procedure.

In all, high-resolution computed tomography is a powerful imaging technique that preserves true 3D-geometry of a specimen and could be seen as a supplementary tool for histology for questions with intermediate resolutional requirements. The diagnostic process profits from the digital data-format which guarantees high accessibility for all involved physicians as well as from fast and precise access to destruction-free volumetric information. Ease of handling and first promising results in the field of advanced soft-tissue staining suggest that the method is ready for larger scale testing of eyes with different pathologies. This could be performed in a routine setting complementary to conventional histology.
